# Supplementary material for: Metabolomics Point out the Effects of Carfilzomib on Aromatic Amino Acid Biosynthesis and Degradation
Source: Int J Mol Sci. 2023 Sep 12;24(18):13966. doi: 10.3390/ijms241813966 (PMC10530946; doi:10.3390/ijms241813966)
Supplement: Supplementary file 1 [file ijms-24-13966-s001.zip › ijms-2596251-supplementary.pdf]

Sample Preparation

The sample preparation was the shame to the previous published protocol[1]:

This study employed plasma, kidney and urine samples of twelve male C57Bl/6J (13-14 weeks of age) mice. The laboratory animals were bred and housed in the Animal Facility of Biomedical Research Foundation Academy of Athens. All in vivo experiments were carried out in accordance with the "Guide for the care and use of Laboratory animals" and experiments were approved by the Ethics Committee (Approval No: 182464;14-05-2019). The mice were housed and maintained according to the ARRIVE guidelines[2].. The animals were randomized in two groups (n=6 for each group) as follows: i) Control (NaCl 0.9%), ii) Cfz (8mg/kg) for 6 days[3]. NaCl and Cfz were injected intraperitoneally on alternate days and at the end of the experiments mice were euthanized by high dose of ketamine (100mg/kg) and subsequent cervical dislocation. Mice were placed in metabolic cages for 24h for urine collection, and they were provided with food and water ad libitum. Plasma samples were collected by centrifugation of heparinized whole blood at 5000 RPM for 15 min. The bio-samples (plasma, kidneys, urine) were collected during and at the end of the experiments and stored at -80°C. Carfilzomib regimens were based on our previous study addressing its cardiotoxicity and are translationally equivalent to human doses[3]. Briefly, in humans Cfz initial dosing is selected to be 27 or 56 mg/m^2 and can be reduced to 15 mg/m^2 upon manifestation of life-threatening cardiorenal adverse events, before discontinuation of the therapy. In a translational scope, the dose regimen selected for the four-dose protocol is equivalent to a HED of 29.65 mg/m2, which is within the range of the initiation dose of Carfilzomib.

Samples from three tissues, i.e., plasma, kidney, and urine were employed. Different experimental protocols were implemented for the extraction of metabolites from each sample type. In order to avoid the discrimination of some metabolite classes, the sample pre-treatment protocol involved only a protein precipitation step.

Plasma extraction procedure: 600 μL of frozen methanol were added in 200 μL of sample and mixed by vortexing for 20 s and consequently, centrifuged using a NEYA 16R centrifugation apparatus (REMI, Mumbai, India) at 10,000 rpm, 5 min, 4°C. A 350 μL aliquot of the supernatant was evaporated to dryness by a HyperVAC-LITE centrifugal vacuum concentration (Hanil Scientific Inc, Gimpo, Korea). Samples were stored at -80 °C and reconstituted before the analysis with 150 μL of IS mix solution[4,5,6,7].

Urine extraction procedure: 500 μL of sample were centrifuged (10000 rpm, 5 min, 4°C) to precipitate particles. The supernatant was diluted with 1000 μL of a methanol – water solution (1:1 v/v) and an aliquot of 600 μL was evaporated to dryness, stored at -80°C and reconstituted with 150 μL of IS mix solution[6,8,9,10]. The acquired data were corrected using the total volume of excreted urine of each mouse.

Kidney extraction procedure: Kidneys were weighted and mixed with appropriate volume of a methanol – water solution (1:1 v/v) adjusted to sample's weight- for every 100 mg of tissue 1000 μL of solution were added. The sample was homogenized using the tissue homogenizing CKMix lysing kit (Bertin Corp.) and the CRYOLYS EVOLUTION tissue homogenizer (Bertin Instruments, Rockville, USA). Homogenization was accomplished in two rounds: initially the sample tissue with the half of the aforementioned solution was submitted to the "hard" mode of the homogenizer, then the blend was centrifuged at 10,000 rpm, for 10 min and the supernatant was placed in a 10 mL falcon. The rest of the solution was added in the

homogenizing tube with the tissue remainder and submitted to a second cycle of a “soft” mode homogenization. After centrifugation, the supernatant was mixed with the one obtained by the first homogenization cycle and vortexed for 10 s. An aliquot of 500 µL of the total extract were evaporated until dryness, stored at -80 °C and reconstituted with 150 µL of IS mix solution before the LC-MS analysis.

Heatmap representation for up/down regulation

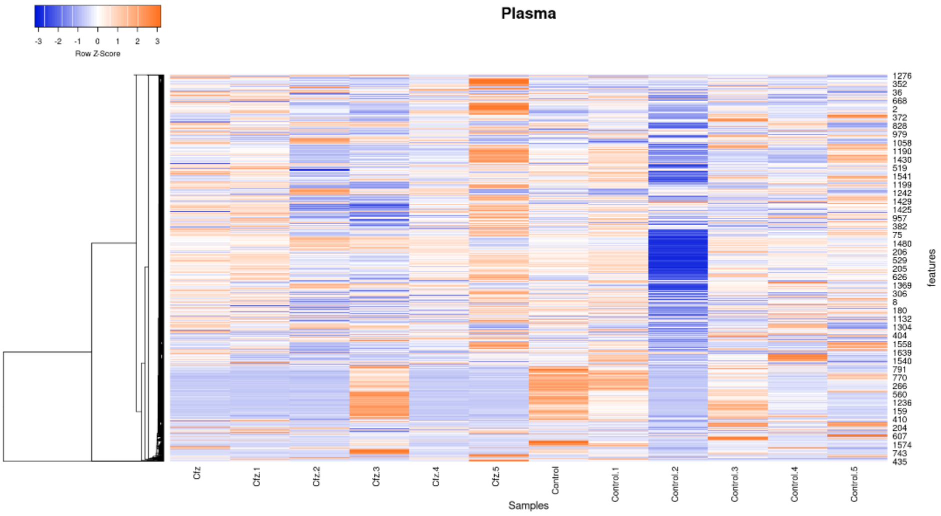

Figure S1. Heatmap representation of metabolites regulation if the plasma (+) dataset

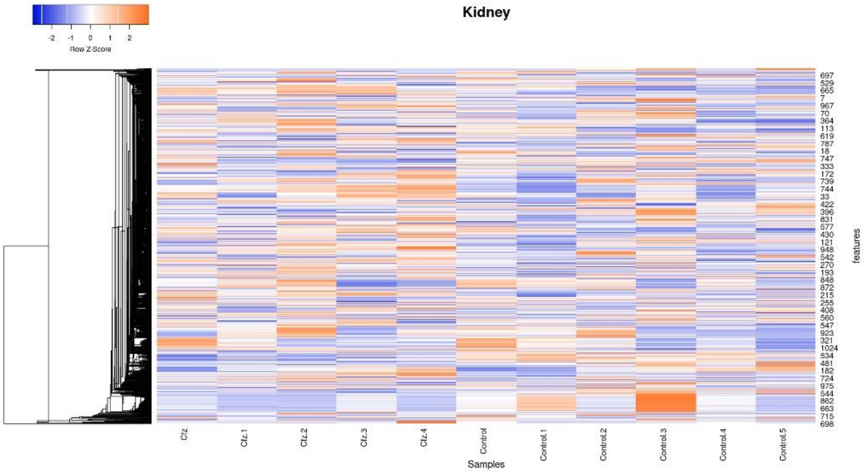

Figure S2. Heatmap representation of metabolites regulation if the kidney (+) dataset

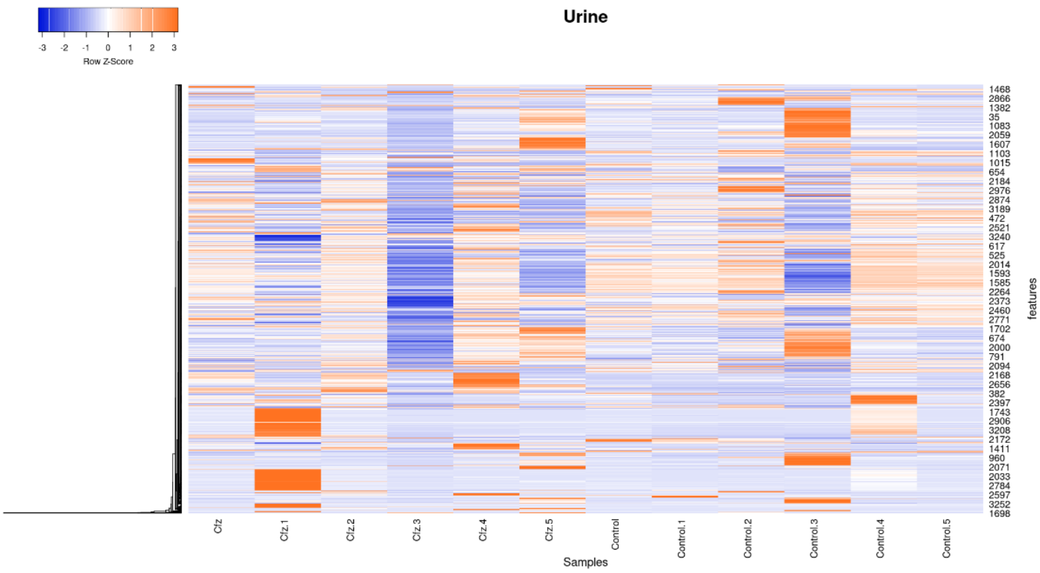

Figure S3. Heatmap representation of metabolites regulation if the urine (+) dataset

Literature

1. Barla I, Efentakis P, Lamprou S, et al. An Untargeted Metabolomics Approach on Carfilzomib-Induced Nephrotoxicity. *Molecules*. 2022;27(22). doi:10.3390/molecules27227929

2. Kilkenny C, Browne WJ, Cuthill IC, Emerson M, Altman DG. Improving bioscience research reporting: The arrive guidelines for reporting animal research. *PLoS Biol*. 2010;8(6). doi:10.1371/journal.pbio.1000412

3. Efentakis P, Kremastiotis G, Varela A, et al. *Molecular Mechanisms of Carfilzomib-Induced Cardiotoxicity in Mice and the Emerging Cardioprotective Role of Metformin*.; 2019. http://ashpublications.org/blood/article-pdf/133/7/710/1552545/blood858415.pdf.

4. Jiang F, Liu Q, Li Q, et al. Signal Drift in Liquid Chromatography Tandem Mass Spectrometry and Its Internal Standard Calibration Strategy for Quantitative Analysis. *Anal Chem*. 2020;92(11):7690-7698. doi:10.1021/acs.analchem.0c00633

5. Yuan M, Breitkopf SB, Yang X, Asara JM. A positive/negative ion-switching, targeted mass spectrometry-based metabolomics platform for bodily fluids, cells, and fresh and fixed tissue. *Nat Protoc*. 2012;7(5):872-881. doi:10.1038/nprot.2012.024

6. Dunn WB, Broadhurst D, Begley P, et al. Procedures for large-scale metabolic profiling of serum and plasma using gas chromatography and liquid chromatography coupled to mass spectrometry. *Nat Protoc*. 2011;6(7):1060-1083. doi:10.1038/nprot.2011.335

7. Wawrzyniak R, Kosnowska A, Macioszek S, Bartoszewski R, Markuszewski MJ. New plasma preparation approach to enrich metabolome coverage in untargeted metabolomics: Plasma protein bound hydrophobic metabolite release with proteinase K. *Sci Rep*. 2018;8(1):1-10. doi:10.1038/s41598-018-27983-0

8. King AM, Mullin LG, Wilson ID, et al. Development of a rapid profiling method for the analysis of polar analytes in urine using HILIC–MS and ion mobility enabled HILIC–MS.

Metabolomics. 2019;15(2):1-11. doi:10.1007/s11306-019-1474-9

9. Wang X, Zhang A, Han Y, et al. Urine metabolomics analysis for biomarker discovery and detection of Jaundice syndrome in patients with liver disease. *Mol Cell Proteomics*. 2012;11(8):370-380. doi:10.1074/mcp.M111.016006

10. Want EJ, Wilson ID, Gika H, et al. Global metabolic profiling procedures for urine using UPLC-MS. *Nat Protoc*. 2010;5(6):1005-1018. doi:10.1038/nprot.2010.50
